# Medicare cost of colorectal cancer screening: CT colonography vs. optical colonoscopy

**DOI:** 10.1007/s00261-015-0538-1

**Published:** 2015-09-09

**Authors:** Bruce Pyenson, Perry J. Pickhardt, Tia Goss Sawhney, Michele Berrios

**Affiliations:** Milliman, One Pennsylvania Plaza, 38th Floor, New York, NY 10019 USA; University of Wisconsin School of Medicine & Public Health, Madison, WI USA

**Keywords:** CT colonography, Optical colonoscopy, Medicare, Costs, Cost-effectiveness

## Abstract

*Purpose*: To compare the Medicare population cost of colorectal cancer (CRC) screening of average risk individuals by CT colonography (CTC) vs. optical colonoscopy (OC). *Methods*: The authors used Medicare claims data, fee schedules, established protocols, and other sources to estimate CTC and OC per-screen costs, including the costs of OC referrals for a subset of CTC patients. They then modeled and compared the Medicare costs of patients who complied with CTC and OC screening recommendations and tested alternative scenarios. *Results*: CTC is 29% less expensive than OC for the Medicare population in the base scenario. Although the CTC cost advantage is increased or reduced under alternative scenarios, it is always positive. *Conclusion*: CTC is a cost-effective CRC screening option for the Medicare population and will likely reduce Medicare expenditures for CRC screening.

**Electronic supplementary material:**

The online version of this article (doi:10.1007/s00261-015-0538-1) contains supplementary material, which is available to authorized users.

The United States Preventive Services Task Force (USPSTF) is reevaluating the evidence for colorectal cancer (CRC) screening, including the efficacy of CT colonography (CTC) [1]. The USPSTF will assign a grade of A or B to CTC screening if they find sufficient evidence to substantiate CTC screening which provides a net population health benefit [2]. Based on currently available data, some experts believe that the USPSTF will give an A or B grade to CTC screening [3, 4], which would require private health insurance plans to cover CTC screening [5]. Medicaid plans would also be required to cover CTC screening for Affordable Care Act “expansion adult” enrollees [6] and for all enrollees if the state has an extra federal match for USPSTF preventative services [7]. While Medicare often follows USPSTF’s lead, Medicare is not required to cover USPSTF A and B services and may make its own coverage decision [8].

Medicare policy has been guided by the “triple aim” since 2010 [9]. The triple aims are to

1. Improve the patient experience of care (including quality and satisfaction),
2. Improve the health of populations, and
3. Reduce the per capita cost of health care [10].

Providing Medicare enrollees access to CTC for CRC screening should improve patient adherence to American Cancer Society (ACS) CRC screening guidelines and patient satisfaction [3, 11, 12]. CTC offers distinct advantages for Medicare enrollees compared to optical colonoscopy (OC or simply “colonoscopy”): CTC is less invasive, has fewer complications, and needs no anesthesia. No anesthesia avoids the need for an escort for the patient post-procedure. Medicare coverage of CTC is therefore consistent with the first two goals of the triple aim.

This paper addresses cost, the third goal of the triple aim. Officially, Medicare does not consider cost in its coverage determinations. However, the prominence of the triple aim, the push toward “value-based care” [13], budgetary stress, and analysis of recent coverage decisions have led some to conclude that cost is likely a factor in Medicare coverage decisions [14]. Furthermore, private Medicare Advantage plans now cover over 30% of Medicare enrollees [15], and they are permitted to cover services not covered by Medicare [16]. Cost is critically important for private payer plans.

The last published evaluation of the relative cost of CTC to OC for Medicare was prepared in 2009 using Medicare OC fees from 2007 [17]. With substantial changes in reimbursement and clinical practices in recent years and additional evidence demonstrating similar efficacy of CTC and OC, this paper fills an important gap.

## Methods

We estimated the per-screen costs of OC and CTC, the frequency of colonic and extra-colonic screening findings and the resulting rescreen times, the size and demographic mix of the Medicare population, and built a simulation model to produce Medicare population-level cost comparisons of the two screening methods. In addition, we tested several alternative scenarios. Throughout this paper, “costs” refers to Medicare allowed amounts, which include the Medicare payment and the enrollee cost sharing payment. For bowel preparation agents, the allowed amounts are those administered by the Medicare Part D insurer.

### OC per-screen costs

We used the 2013 Medicare 5% Sample of Medicare Part A and Part B enrollment and claims as our primary data source for OC costs [18]. We adjusted the claim costs for changes in Medicare fee schedules between 2013 and 2015 and, because the 5% Sample does not contain prescription drug data, separately developed costs for bowel preparation agents. To include all colonoscopy-related costs, we collected all costs for the day of a colonoscopy (a “colonoscopy day”) and excluded the costs clearly not related to the colonoscopy. We separately quantified the cost of colonoscopies with and without biopsies.

We limited the OC cost analysis to Medicare enrollees with Medicare Part A and Part B coverage. We excluded Medicare Advantage and other capitated enrollees as claims are unreliable [19]. We identified an enrollee as having a colonoscopy day by the presence of a non-inpatient professional or technical claim with an allowed charge greater than $0 and a Healthcare Common Procedure Coding System (HCPCS) code or Current Procedural Terminology (CPT^®^) code for a colonoscopy (Table 1).

**Table 1:** Colonoscopy codes

		Code indicates	
HCPCS/ CPT^®^ code	Code description	Screening	Biopsy
G0121^a^	Colorectal cancer screening; colonoscopy on individual not meeting criteria for high risk	Always	No^c^
G0105^a^	Colorectal cancer screening; colonoscopy on individual at high risk	Always	No^c^
45378	Colonoscopy, flexible, proximal to splenic flexure; diagnostic, with or without collection of specimen(s) by brushing or washing, with or without colon decompression (separate procedure)	Maybe^b^	No^c^
45379	Colonoscopy, with removal of foreign body	Maybe^b^	No^c^
45380	Colonoscopy, with biopsy, single or multiple	Maybe^b^	Always
45381	Colonoscopy, with directed submucosal injection(s), any substance	Maybe^b^	Always
45382	Colonoscopy, with control of bleeding (e.g., injection, bipolar cautery, unipolar cautery, laser, heater probe, stapler, plasma coagulator)	Maybe^b^	Always
45383	Colonoscopy, with ablation of tumor(s), polyp(s) or other lesion(s) not amenable to removal by hot biopsy forceps, bipolar cautery, or snare technique	Maybe^b^	Always
45384	Colonoscopy, with removal of tumor(s), polyp(s), or other lesion(s) by hot biopsy forceps or bipolar cautery	Maybe^b^	Always
45385	Colonoscopy, with removal of tumor(s), polyp(s), or other lesions by snare technique	Maybe^b^	Always
45386	Colonoscopy, with dilation by balloon, 1 or more strictures	Maybe^b^	No^c^
45387	Colonoscopy, with transendoscopic stent placement (includes predilation)	Maybe^b^	No^c^
45391	Colonoscopy, with endoscopic ultrasound examination	Maybe^b^	No^c^
45392	Colonoscopy, with transendoscopic ultrasound-guided intramural or transmural fine-needle aspiration/biopsy(s)	Maybe^b^	No^c^

^a^Code is used only by Medicare, specifically for screening colonoscopies

^b^If accompanied by an ICD-9-CM diagnosis code of V7651 (screen for malignant neoplasm-colon), a procedure modifier code of PT (colorectal cancer screening test, converted to a diagnostic test or other procedure), or a procedure modifier code of 33 (preventative service)

^c^A separate same-day claim for CPT code 88305 (Level IV—Surgical pathology—colon biopsy, Lymph node biopsy, Colorectal Polyp) indicates a biopsy

Most colonoscopies have both professional and technical claims. We classified the colonoscopy day as screening if we found either a professional and/or a technical claim with a HCPCS code indicating screening, a diagnostic colonoscopy CPT code accompanied by a diagnosis code indicating screening, or a procedure modifier code indicating either a screening or a preventive service. We excluded colonoscopy days with procedure modifiers indicating reduced services or an incomplete procedure (less than 2% of colonoscopy days). See Table 1.

We classified a colonoscopy day as having a biopsy if there was a same-day professional and/or technical claim that indicated a biopsy, or we found one or more same-day colon biopsy pathology claims (Table 1). More than 95% of colonoscopies coded as a biopsy were accompanied by a pathology claim. We classified a colonoscopy day as having an anesthesia service if we identified a same-day professional outpatient anesthesia claim.

We excluded somewhat more than 10% of the screening colonoscopy days because they included both colonoscopies and upper endoscopies. We excluded colonoscopy days for patients under age 50 (less than 2%). Within a colonoscopy day, we excluded costs not directly relevant to screening colonoscopies, including inpatient services, emergency room services, prescription drugs, and durable medical equipment, totaling less than 2% of the total costs. A portion of these costs may be relevant to screening complications. We later tested for the potential cost impact of complications.

Colonoscopy bowel preparation costs (cathartic agents) are not covered under Medicare Part A or Part B but are covered under Medicare Part D. About 67% of Medicare beneficiaries with Parts A and B also have Part D. Separate, detailed Part D spending data were recently released [20]. Within the Part D data, we examined the cathartic agents prescribed by gastroenterologists and found the average cost for the American Society for Gastrointestinal Endoscopy (ASGE) recommended polyethylene glycol (PEG)-based preparations, such as MoviPrep and GaviLyte, and sodium phosphate-based preparations such as Suprep [21]. We note that some physicians recommend OTC cathartic agents that cost less than these agents and are not paid for by Medicare Part D [22].

In order to project the historical 2013 data to reflect 2015 cost levels, we calculated a weighted average 2013 to 2015 Medicare cost trend for the most frequent colonoscopy HCPCS (or CPT) codes, by site of service, using Medicare fee schedules. We applied the 2013 site of service distribution derived from colonoscopy claims identified in the Medicare 5% Sample, and estimated the per-unit colonoscopy costs for 2015.

### CTC per-screen costs

Since Medicare currently does not have a fee for screening CTC, we used the current Medicare fee schedule for diagnostic CTC without intravenous (IV) contrast (CPT code 74261) for screening CTC. Since CTC requirements are similar for screening and non-contrast diagnostic purposes [23], we expect that Medicare reimbursement will be the same for a screening CTC as it is for a diagnostic CTC without IV contrast. Under Medicare’s fee system, the fee for diagnostic CTC has two components: professional and technical. The Medicare Physician Fee Schedule (MPFS) for the professional services applies across all sites of service. Technical service fees may be, however, site specific. While the technical component CTC fee for physician offices/clinics per the MPFS is substantially higher than for hospital outpatient departments (HOPDs), the Outpatient Prospective Payment System (OPPS), Section 5102(b) of the Deficit Reduction Act of 2005 limits payment for the technical component of imaging services to the lesser of the MPFS or the OPPS fees [24, 25]. Therefore, the 2015 national Medicare fee for diagnostic CTC is the same across settings (unlike colonoscopy).

Guidelines recommend that CTC bowel prep includes the use of cathartic and oral contrast tagging agents [23, 26, 27]. The recommended cathartic agents are the same as those for OC bowel preparation. Oral contrast tagging agents are included in the CTC fee, and no extra amounts for the tagging agents are billable to Medicare or the patient.

According to the American College of Radiology (ACR) and ACS Guidelines [23, 28], CTC patients with polyps 6+ mm should be offered follow-up OC with polypectomy. For patients with follow-up OC, we added the colonoscopy with biopsy fees to the CTC costs.

### Screening findings, OC follow-up, and rescreen times

The size of the largest polyp is the most important finding for determining the CRC rescreen times and whether a CTC patient will have follow-up OC. The commonly reported size categories are 5 mm or smaller (diminutive), 6–9 mm (small), and 10 mm and larger (large). Other relevant characteristics for OC polyp findings include the total number of polyps, carcinoma status, adenoma status, hyperplasia status and grade, and other descriptors (hyperplastic, tubular, sessile, and serrated) [29]. The current OC standard of care, suggested by the ACS [30] and American College of Gastroenterology (ACG) [31] guidelines and encouraged by quality of care measures for adenoma detection rate and polypectomy rate [32, 33], is for physicians to remove all polyps, irrespective of size.

We combined the probability of large polyps reported by Lieberman [34] with the relative risk factor for small and diminutive polyps as reported by Lowenfels [35] to produce a table of polyp size findings by age and sex. The Lieberman and Lowenfels studies used data centered at approximately 2005. The literature suggests that colonoscopists are being asked to find and remove even diminutive polyps [32, 33, 36]. To account for increased polyp detection rates in recent years and Medicare’s demographics, we used Lieberman’s probability for large polyps, Lowenfels’ probability of small polyps relative to large polyps, and the results of our Medicare 5% Sample analysis for the probability of any polyp, with the probability of a diminutive polyp as the difference between the probability of any polyp and the sum of the probabilities of large and small polyps. See the Appendix in supplementary material for sample calculations.

We used the same large and small polyp detection rates for both OC and CTC. We ignored diminutive polyps potentially identified by CTC as they are not reported in isolation and do not impact rescreen time [23]. The literature indicates that CTC screenings may be either as sensitive or somewhat less sensitive as OC screenings for large and small polyp detection [37]. Assuming less sensitivity for CTC would lower CTC costs as fewer patients would be offered OC.

For OC, the consensus guidelines of the US Multi-Society Task Force on Colorectal Cancer as published by the AGA Institute recommend rescreening of average risk individuals^1^ in 10 years if no polyps are found and, except for rare findings,^2^ between 3 and 10 years if polyps are found. Specific recommendations depend on the number of polyps and their characteristics [29]. We modeled estimates of the number of polyps and their characteristics in order to estimate the average, guideline based, years to OC rescreen.

The CTC guidelines of the ACR [23], the ACS [28], and the US Multi-Society Task Force on Colorectal Cancer recommend that patients with polyps 6+ mm (small and large) are to be offered follow-up OC [29]. The ACS guideline acknowledges that some patients with small polyps will opt for surveillance. We assumed that 100% of the CTC patients with large polyps and 50% of patients with small polyps will have follow-up OC. The 50% assumptions are in the range reported informally by practicing CTC radiologists, although given the lack of published data, we tested alternative rates via scenario testing.

The CTC guidelines of the ACG and the ACS recommend CTC every 5 years, and the Working Group on Virtual Colonoscopy recommends CTC every 5–10 years [23, 30, 31, 38]. Since the ACR [39] does not recommend reporting diminutive polyps, we assume a 5-year interval for individuals with no polyps or diminutive polyps. The CTC guidelines are not specific, however, for the rescreening intervals of patients with previous small or large polyps. We assumed that the rescreen interval will be the minimum of the OC rescreen interval or 5 years.

We assumed CRC screening would start at age 50, which is consistent with the guideline recommendations of the USPSTF [40], the ACS [30], and the ACG [31] for most average risk individuals.^3^ The guidelines, however, are inconsistent for the maximum age for screening. USPSTF recommends through age 74 for most individuals. The American Gastroenterological Association (AGA) Institute and the ACG are silent with respect to stop age and, as a payor, Medicare sets no upper age limit [41]. Although we observed a decline in Medicare screening colonoscopy rates after age 74, 19% of screening colonoscopies were for patients age 75–84 and 1% of colonoscopies for patients age 85 and over. We set the start and stop ages for our base scenario at ages 50 and 85, meaning that everyone receives their first screening at age 50 and no one has a screening age 85 and older.

### Medicare population

CRC screening is covered under Medicare Part B. We estimated the size of the 2015 Medicare Part B population by age and sex by adjusting the Medicare Part B enrollment from the 2013 Medicare 100% Sample [18] by the ratio of 2015 [42] and 2013 [43] population estimates. We estimated the size of the entire Medicare Part B population. Medicare indirectly pays for the cost of CRC screening for Medicare Advantage enrollees via Medicare Advantage capitation payments, and such plans must cover all Medicare covered services.

### Simulation model

To examine the cost differences between OC and CTC, we built a simulation model that follows a population of individuals at annual increments starting at age 50 through their screening years on an OC or a CTC path. The model assumed perfect compliance with the screening intervals specified for each scenario, without cross over between the two paths. The model used random numbers and the probabilities described in Methods and Results to determine CTC and OC findings, whether an OC patient has a biopsy, and whether a CTC patient with a small polyp has a follow-up OC. The screenings were monetized using the costs described.

We simulated a population of 10,000 individuals for each path. We weighted the resulting age-sex-specific costs and other results by the 2015 Medicare age-sex distribution to calculate Medicare population totals. We assumed no cost inflation and no discounting. The results are 2015 costs as if all 2015 Medicare enrollees had been on either a CTC or OC screening path since age 50.

We used SAS Version 9.3 for data analysis and Microsoft Excel 2010 for the simulation modeling.

## Results

We identified 127,175 Medicare colonoscopies performed in 2013 (Table 2) and classified 56,578 (44%) as screening for purposes of calculating average costs. 46% of the colonoscopies were excluded because the coding indicated that they were diagnostic, 7% because they were performed on the same-day as an upper endoscopy, 2% because they were incomplete, and 1% because the Medicare enrollee was under age 50.

**Table 2:** 2013 Medicare colonoscopies

Colonoscopy days identified	Number	Percentage
Total	127,175	100
Exclusions:		
Diagnostic	58,206	46
Incomplete	2441	2
With same-day upper endoscopy	9139	7
Enrollee under age 50	811	1
Net remaining: screening colonoscopy days	56,578	44

*Source*: Authors’ analysis of 2013 Medicare 5% sample data

We estimated 2015 average Medicare colonoscopy screening costs of $1,035 (Table 3), using trends estimated by comparing 2013 and 2015 Medicare fees. The average cost was higher for colonoscopies with biopsies ($1212) and lower for colonoscopies without biopsies ($824). Most of the cost difference was due to the professional and technical fees for the colonoscopy and not pathology costs. Pathology costs averaged $92 for a colonoscopy with biopsy. 54% of colonoscopies have a biopsy. Costs varied depending on the colonoscopy codes, whether the colonoscopy involved separately billed anesthesia, the site of the colonoscopy (outpatient hospital, ambulatory surgical center, or physician office), the number of biopsies when there is a biopsy, the bowel preparation agent, and the providers’ locality. Under Medicare rules, twilight sedation administered by the colonoscopist and staff is not separately billed [44]. Deep sedation, typically propofol, must be administered by an anesthesiology professional and is separately billed [45]. 57% of colonoscopies had separately billed anesthesia costs. The average anesthesia cost per colonoscopy with a separately billed anesthesia cost was $154 ($88/57%). Bowel preparation agents were $39 of the total cost and reflect the discount that Medicare Part D plans receive compared to average wholesale prices.

**Table 3:** 2013 and 2015 Medicare average costs for screening colonoscopies

Screening colonoscopy days	Without biopsy	With biopsy	Total/avg
N	25,850	30,728	56,578
% *n*	46%	54%	100%
Actual 2013 costs per screening			
Professional and technical	$672	$949	$822
Anesthesia^a^ (separately billed)	85	91	88
Pathology^a^	0	92	50
Bowel preparation agents	39	39	39
Total	797	1171	1000
Estimated 2015 costs per screening	$824	$1212	$1035

^a^Pathology and anesthesia costs are averaged across colonoscopies with and without these costs

*Source*: Authors’ analysis of 2013 Medicare 5% Sample data, 2013 Part D prescription drug data, and (for trend) Medicare 2013 and 2015 fee schedules

Medicare’s 2015 national fee schedule for diagnostic CTC without IV contrast is $243 (Table 4) of which $123 is professional and $120 is technical. We assumed that total 2015 cost for a screening CTC is the cost of the diagnostic CTC, the cathartic bowel preparation agent (using same cost as for OC bowel preparation—Table 3), and the cost of an OC with biopsy (Table 3) for the patients with follow-on OC. The oral contrast tagging agent(s) bowel preparation agents used specifically for CTC (in addition to the cathartic agent) is included in the Medicare fee and not separately billed.

**Table 4:** 2015 Medicare diagnostic CTC cost

Diagnostic CTC (CPT-4 code 74261)	Medicare fee schedule
Professional component	$123
Technical component (after payment limit)	$120
Professional and technical^a^	$243

^a^Oral contrast tagging agent costs are included in professional and technical fee

*Source*: http://www.cms.gov/apps/physician-fee-schedule/, April, 2015

Using the data sources and assumptions described in Methods and further described in the Appendix in supplementary material, we estimated that colonoscopies for male Medicare enrollees’ ages 65–74 (the decade with the highest Medicare enrollment) have approximately 10%, 12%, and 38% probabilities finding large, small, or diminutive polyps, respectively (Table 5). Probabilities for large and small polyps generally increase by age while probabilities for diminutive polyps peak at ages 60–64. Probabilities for large and small polyps were consistently lower for females than for males but were similar for diminutive polyps.

**Table 5:** Probabilities of OC and CTC findings

	Size of largest polyp				
Ages	None (%)	<6 mm (diminutive) (%)^a^	6–9 mm (small) (%)	10+ mm (large) (%)	All (%)
Male					
50–54	48.4	36.9	8.6	6.1	100
55–59	44.4	38.0	10.3	7.3	100
60–64	38.7	42.1	10.6	8.6	100
65–69	41.0	37.6	11.8	9.6	100
70–74	40.3	38.7	11.3	9.7	100
75–79	39.3	38.4	12.0	10.3	100
80–84	44.0	33.4	11.6	11.0	100
Female					
50–54	50.4	39.2	6.4	4.0	100
55–59	47.4	40.6	7.4	4.6	100
60–64	48.1	39.4	7.3	5.2	100
65–69	50.9	35.2	8.1	5.8	100
70–74	49.4	35.7	8.5	6.4	100
75–79	48.9	34.6	9.4	7.1	100
80–84	49.4	34.6	8.7	7.3	100

^a^Not used for the CTC analysis other than for scenario 9; isolated diminutive polyps identified by CTC are not reported and as a finding has the same effect as no polyps

*Source*: Authors’ analysis of published literature and 2013 Medicare 5% Sample data. See Appendix in supplementary material

Guideline recommendations imply that screening OC patients with diminutive and small polyps should be rescreened on average in 7 or 6 years, respectively. Table 6 shows the guideline and estimated average years until rescreening, rounded to the nearest integer year, for all OC and CTC polyps sizes. The literature indicates that rescreens often occur sooner than recommended by guidelines [46]; scenario 8 tests the impact of more frequent rescreens.

**Table 6:** Years to rescreen based on OC and CTC findings

			Size of largest polyp			
Screen	Ages	Sex	None	<6 mm (diminutive)	6–9 mm (small)	10+ mm (large)
OC	All	Both	10	7	6	3
CTC	All	Both	5	5	5	3

*na* Not applicable

*Source*: For OC, Authors’ analysis of US Multi-Society Task Force on Colorectal Cancer Guidelines [29] and published literature. For CTC, the lesser of 5 years or the rescreen time for OC

As of 2015, there are nearly 43 million Medicare enrollees’ ages 50–84 with Part B or Part C coverage, which provides payment for colorectal cancer screening. Nearly ½ (45.8%) of the enrollees are of ages 65–74 and the majority of those are of ages 65–69. Table 7 summarizes our estimated 2015 Medicare population by sex and 5-year age bands. The simulation applies the screening and follow-up criteria to this population by sex and single year age bands.

**Table 7:** 2015 Medicare population (000s)

	Male		Female		Total	
Ages	Enrollees	%	Enrollees	%	Enrollees	%
Under 50	1511	6.7	1353	4.8	2864	5.6
50–54	729	3.2	698	2.5	1427	2.8
55–59	957	4.2	945	3.4	1902	3.7
60–64	1632	7.2	1759	6.2	3391	6.7
65–69	6005	26.5	7114	25.2	13,119	25.8
70–74	4628	20.4	5550	19.7	10,178	20.0
75–79	3254	14.4	4139	14.7	7393	14.5
80–84	2167	9.6	3122	11.1	5289	10.4
85+	1788	7.9	3508	12.4	5296	10.4
Total all ages	22,670	100.0	28,189	100.0	50,860	100.0
Total ages 50–84	19,372	85.4	23,328	82.8	42,699	84.0

*Source*: Authors’ analysis of 2013 Medicare 100% sample and US Census Bureau data

Table 8 summarizes the population-level statistics for the base simulation. If all of the 2015 Medicare enrollees had adhered with either OC or CTC screening guidelines (paths) since the age of 50, we estimate that there would be 5.5 million OCs or 9.1 CTCs performed in 2015. The volume difference is the result of an average time to rescreen of 8.0 years for OC and 4.9 years for CTC.

**Table 8:** Base simulation summary statistics for the 2015 Medicare population

	Screening path	
Statistic	OC	CTC
2015 total screenings (000s)	5501	9159
Male %	46.5%	45.8%
Female %	53.5%	54.2%
Screenings findings by size of largest polyp		
None	46.3%	45.9%
<6 mm (diminutive)	37.3%	37.4%
6–9 mm (small)	9.1%	9.5%
10+ mm (large)	7.3%	7.2%
Any size	53.7%	54.1%
6+ mm	16.4%	16.7%
CTC with follow-up OC %	na	12.9%
Average cost per screening	$1036	$439
Average years to rescreen	8.0	4.9

*na* Not applicable

*Source*: Authors’ simulation. Assumes all Medicare enrollees have perfectly adhered to an OC or CTC screening path since age 50

The higher volume of CTC screenings is offset by a substantially lower price. The average OC screening costs $1,036 and the average CTC screening costs $439, inclusive of the follow-up OC costs for 12.9% of the CTC screens that receive follow-up OC. We estimated that fully screening all Medicare enrollees charged $5.7 billion for the OC path and $4.0 billion for the CTC path, $9.34 per Medicare enrollee per month for OC and $6.59 for CTC. CTC is 29% less costly (Table 9).

**Table 9:** Base simulation results for the 2015 Medicare population

	Screening path	
Result	OC	CTC
2015 total cost ($000s)	$5,699,109	$4,023,988
Cost per Medicare enrollee per month^a^	$9.34	$6.59
Cost per screening age Medicare enrollee per month^b^	$11.12	$7.85
CTC savings compared to OC	29%	

^a^Denominator is 2015 total Medicare population

^b^Denominator is the 2015 Medicare population ages 50–84

*Source*: Authors’ simulation. Assumes all Medicare enrollees have perfectly adhered to an OC or CTC screening path since age 50

## Scenario tests and results

To understand the model sensitivity to certain assumptions and potential changes to OC and CTC standards and practices, we tested several alternative scenarios. Table 10 describes the scenarios (with more detail provided in the Appendix in supplementary material) and explains why we selected them. Table 11 summarizes the results of the scenarios.

**Table 10:** Alternative scenarios

Description	Alternative model inputs	Explanation
1. Fewer large and small polyps	Apply a 0.80 adjustment factor to the probability of large and small polyps and increase the probability of diminutive size polyps by the same amount so that the probability of finding a polyp of any size remains unchanged	Our base scenario, using data centered at approximately 2005, produces an approximately 18% aggregate probability of a large or small polyp finding. We have found smaller studies that indicate an aggregate probability in the 13–15% range [47–49]. Since the probability of finding a polyp of any size, however, uses recent data we left the total probability unchanged
2. Add costs for OC and CTC complications and CTC extra-colonic findings	OC costs: add $20 and $96 to OC without and with biopsy costs, respectively, for OC complications	We used published literature [50], trended to 2015, to estimate the costs of complications and the follow-up diagnosis costs of CTC extra-colonic findings. OC has more complications than CTC and within OC, OCs with biopsies have substantially more complications than OCs without biopsies [47, 49, 51]. See Appendix in supplementary material for more details. The ACR currently recommends the reporting of potentially significant extra-colonic findings [23]
	CTC costs: add $131 to CTC for CTC complications and extra-colonic findings	
3. Increase anesthesia use for OC	Assume that 80% of OCs will have separately billed anesthesia, a 40% increase in use and costs from the 57% base scenario assumption	In 2013 separately billed anesthesia was subject to Medicare cost sharing; as of January 1, 2015 it is no longer subject to cost sharing [52]. Anesthesia use may therefore increase over the next couple of years
4. Add costs for CTC shared decision making	Add a $20 cost to all CTCs and another $20 cost to CTCs with small polyps	Medicare covers CT lung cancer screening with the provision that the first screening must include a documented shared decision making consultation [53]. If Medicare adopts a similar approach for CTC screening, two consultations may be required: the first for the screening and the second for the decision for follow-up OC if the patient has small polyps. See the Appendix in supplementary material for more details
5. Decrease maximum screening age	Decrease maximum screening age from age 84 to age 74	UPSTF recommends CRC screening until age 75. Medicare, however, currently pays for screening for all ages 50 and over
6. Decrease OC follow-up rate for CTCs with small polyp findings	Decrease OC follow-up rate for CTCs with small polyp findings from 50% to 25%	No one knows how many patients with small polyps will opt for OC polypectomy vs. CTC surveillance. The percentage will likely vary substantially by clinic and physician
7. Increase OC follow-up rate for CTCs with small polyp findings	Increase OC follow-up rate for CTCs with small polyp findings from 50% to 75%	
8. Decrease rescreen years for both OC and CTC for screenings with small polyps	Rescreen in 3 years instead of the 6 years for OC and 5 for CTC	Literature indicates that many OC patients rescreen sooner than recommended by guidelines [46]
9. Increase rescreen years for CTC to match OC	Rescreen in 3, 6, 7, and 10 years for large, small, diminutive, and no polyps, respectively	By removing the rescreen time differential, this scenario compares the per-screen costs of CTC and OC

**Table 11:** Alternative scenario simulation results

	2015 cost per Medicare enrollee per month		
	Screening path		CTC savings (%)
Scenario	OC	CTC	
0. Base	$9.34	$6.59	29
1. Fewer large and small polyps	9.31	6.09	35
2. Add costs for OC and CTC complications and CTC extra-colonic findings	9.89	8.74	12
3. Increase anesthesia use for OC	9.68	6.67	31
4. Add costs for CTC shared decision making	9.34	7.00	25
5. Decrease maximum screening age	6.66	4.46	33
6. Decrease OC follow-up rate for CTCs with small polyp findings	9.34	6.13	34
7. Increase OC follow-up rate for CTCs with small polyp findings	9.34	7.03	25
8. Decrease rescreen years for both OC and CTC for screenings with small polyps	9.65	6.80	30
9. Increase rescreen years for CTC to match OC	9.34	3.97	58

*Source*: Authors’ simulation. Assumes all Medicare enrollees have perfectly adhered to an OC or CTC screening path since age 50

Scenarios 1 through 9 produce CTC savings ranging from 12% to 58% compared to the base scenario savings of 29%. The 12% savings (scenario 2) results from including estimates, using data from published research, for the costs of OC and CTC complications and CTC extra-colonic findings. Extra-colonic findings add costs only to CTCs and complications add more costs to OC than CTC. While complications never benefit the patient, CTC extra-colonic findings, such as early detection of other cancers or abdominal aorticaneurysm may benefit the patient.

The cost advantage of CTC to OC diminishes when more CTC patients with small polyps have follow-up OC rather than surveillance or when CTC screenings are accompanied with shared decision making consultations. The impact of increasing from 50% to 75% the CTC patients with small polyps having follow-up OC (scenario 7) is similar to the impact of the shared decision encounter (scenario 4). Both reduce CTC savings by about 4% (25% savings compared to 29% for the base scenario). The cost impact is linear. Therefore, the impact of 100% of CTC patients with small polyps having follow-up OC or $40 shared decision costs is 8% (21% savings compared to 29% for the base scenario).

Scenarios 1, 3, 5, 6, and 9 increase the cost advantage of CTC to OC. Scenario 9 assumes CTC screening at the same intervals as OC. The scenario is included to compare the per-screen costs of OC and CTC without consideration of the costs resulting from more frequent CTC screenings. On a per-screen basis, inclusive of the costs for patients with large and small polyps having follow-up OC, CTC is nearly 60% less costly than OC screening.

## Discussion

For a Medicare population, when compared to OC, CTC satisfies the third goal of the triple aim: reducing the per capita cost of health care. For our base scenario, CTC is 29% less costly than OC per Medicare enrollee. Although the CTC cost advantage is quite variable under the alternative scenarios, the cost advantage is positive for all alternative scenarios (range: 12–58%).

Our methodology assumes perfect enrollee adherence to either an OC or CTC path with no cross over between paths. This assumption facilitates the cost comparison. In reality, some Medicare enrollees will not be screened, others will be screened too often or for too many years, and others will cross between paths [46, 54, 55]. Because it is less invasive, CTC may have higher compliance than OC [11, 12]. We did not consider any of these possibilities in our analysis.

We identified in the historical Medicare data that 2% of colonoscopies were coded as incomplete, as defined by the failure to advance the scope past the splenic fixture [56]. Medicare reimbursement for incomplete colonoscopies is about 1/3 of that for a complete colonoscopy [24]; a subsequent complete colonoscopy is paid at the full rate [56]. With CTC, there is no colonoscope to advance, and a CTC can always visualize the entire colon, although sometimes with suboptimal assessment [57]. As such, CTCs are rarely incomplete in the same sense. Although we did not consider this difference in our modeling, a lower rate of incomplete CTC screenings relative to OC screenings would further favor CTC over OC when comparing quality and cost.

We note that the probabilities we assign to polyp size findings are “memoryless”—findings from the last screening, including findings of adenomatous polyps, do not impact the modeled probabilities of findings for the next screening. If the existence of prior polyps affects the probability of future polyps, or removal of diminutive polyps reduces the future appearance of small and large polyps, then our results would need reconsideration. Empirical data on this issue are quite limited.

Prior cost-effectiveness analyses vary in their reported results but have generally shown that CTC screening is cost-effective especially compared with no screening [58]. When typical non-Medicare charges and extra-colonic findings (e.g., abdominal aortic aneurysms) are considered, the cost-effectiveness of CTC increases further relative to OC [59]. Equivalence of clinical efficacy for colorectal evaluation between CTC and OC is well established [49, 60, 61].

Separate modeling is required for non-Medicare populations. Commercial insurance, Medicaid, other insurer, and direct-bill costs are substantially different and often higher than Medicare costs with inconsistent cost differentials by service. In particular, commercial insurers generally pay much higher fees for anesthesia and OC than does Medicare. Future changes in Medicare fee schedules and payment methodologies, Medicare assigning a screening CTC fee schedule payment different than the current diagnostic CTC fee schedule, or changes in OC or CTC screening guidelines and practices may increase or decrease the cost advantage of CTC over OC.

## Electronic supplementary material

Supplementary material 1 (PDF 99 kb)

## Abbreviations

ACR: American College of Radiology
ACG: American College of Gastroenterologists
ACS: American Cancer Society
AGA: American Gastroenterological Association
ASGE: American Society for Gastrointestinal Endoscopy
CMS: Centers of Medicare and Medicaid Services
CPT®: Current procedural terminology, a registered trademark^®^ of the American Medical Association (AMA)
CRC: Colorectal cancer
CTC: CT colonography
HCPS: Healthcare common procedure coding system
HOPD: Hospital outpatient department
IV: Intravenous
OC: Optical colonography
OPPS: Outpatient prospective payment system
USPSTF: US Preventative Services Task Force

## Footnotes

1 Individuals without a history of colorectal cancer or polyps with high-grade dysplasia.

2 >10 adenomas or serrated polyposis syndrome.

3 The ACG recommends age 45 for African Americans.
