# Supplementary material for: Medicare cost of colorectal cancer screening: CT colonography vs. optical colonoscopy
Source: Abdom Imaging. 2015 Sep 9;40(8):2966–76. doi: 10.1007/s00261-015-0538-1 (PMC4628615; doi:10.1007/s00261-015-0538-1)
Supplement: Supplementary file 1 — Supplementary material 1 (PDF 99 kb) [file 261_2015_538_MOESM1_ESM.pdf]

Supplementary material

Appendix

Medicare cost of colorectal cancer screening: CT colonography vs. optical colonoscopy
Bruce Pyenson, Perry J. Pickhardt, Tia Goss Sawhney, Michele Berrios
Abdominal Imaging, 2015

Note: The appendix provides supplementary materials not included in the print publication of the above article.

<u>Screening findings</u>

We state the following in our article with regard to polyp size findings:

We combined the probability of large polyps reported by Lieberman [34] *with the relative risk factor for small and diminutive polyps as reported by Lowenfels* [35] *to produce a table of polyp size findings by age and sex. The Lieberman and Lowenfels studies used data centered at approximately 2005. The literature suggests that colonoscopists are being asked to find and remove even diminutive polyps* [32, 36, 33]. *To account for increased polyp detection rates in recent years and Medicare's demographics, we used Lieberman's probability for large polyps, Lowenfels' probability of small polyps relative to large polyps, and the results of our Medicare 5% Sample analysis for the probability of any polyp, with the probability of a diminutive polyp as the difference between the probability of any polyp and the sum of the probabilities of large and small polyps.*

Tables A.1-A.4 provide source data used to develop base probabilities of finding polyps by polyp size, age, and sex used in our simulation analysis. Sample calculations are provided in Table A.5.

Table A.1 – Prevalence (%) of Large Polyp(s) (10+mm) in Screening Colonoscopy

Age	Male	Female
50-54	(a) 6.1%	4.0%
55-59	7.3%	4.6%
60-64	8.6%	5.2%
65-69	9.6%	5.8%
70-74	9.7%	6.4%
75-79	10.3%	7.1%
80+	11.0%	7.3%

Source: Lieberman, et al, 2014
Note: Findings based on CORI data (2000-2011), n=327,785

Table A.2 – Colonoscopies by Polyp Size and Age

	Number of Colonoscopies		Ratio of
Age	Small (6-9mm)	Large (10+mm)	Small to Large
50-59	8,433	4,613	(b) 148%
60-69	6,259	3,809	129%
70-79	3,149	1,905	123%
80+	508	326	111%
Total	18,349	13,560	135%

Source: Lowenfels, 2011, CORI data (2002-2007), n=82,641

Table A.3 – Colonoscopies by Polyp Size and Sex

	Number of Colonoscopies		Ratio of
Sex	Small (6-9mm)	Large (10+mm)	Small to Large

Male	11,502	6,982	(c) 129%
Female	7,920	4,258	147%
Total	19,422	11,240	(d) 136%

Source: Lowenfels, 2011, CORI data (2002-2007), n=82,641
Note: Includes ages <50, hence the difference in totals between Tables A.2 and A.3

Table A.4 – Biopsy Rate of Colonoscopies by Age and Sex

Age	Male	Female
50-54	(e) 51.6%	49.6%
55-59	55.6%	52.5%
60-64	61.3%	51.8%
65-69	59.0%	49.1%
70-74	59.8%	50.6%
75-79	60.7%	51.1%
80+	56.0%	50.6%

Source: Authors' analysis of Medicare 5% Sample (2013), n=57,389 (all ages)

Table A.5 – Sample Calculations for Base Probabilities

Age/Sex	No Polyps (1)	Large (10+mm) (2)	Small (6-9mm) (3)	Diminutive (<6mm) (2)
Male 50-54	48.4%	6.1%	8.6%	36.9%

Notes:
(1) = 1 – Biopsy Rate = 1 – (e) = 1 – 51.6% from Table A.4.
(2) = (a) = 6.1% from Table A.1.
(3) = (2) x (b) x (c) ÷ (d) = 6.1% x 148% x 129% ÷ 136% from Tables A.2 and A.3.
(4) = 1 – (1) – (2) – (3) = 1 – 48.4% – 8.6% – 6.1%

Scenario testing

In addition to the base scenario, we tested several alternative scenarios, described in Table 10 of the article. We provide additional detail for all scenarios below.

Alternative scenario 1 – fewer large and small polyps

The base scenario assumes an aggregate probability of finding large (10mm and larger) or small (6-9mm) polyps of about 18%, based on literature using sample data centered at approximately 2005. More recent studies using smaller sample size suggests that the probability of finding large or small size polyps has decreased. To test these more recent findings we reduced the probabilities of finding large and small size polyps by 20% for an aggregate probability of finding large or small polyps of about 14%. In addition, we increased the probability of diminutive size polyps by the same absolute amount so that the probability of finding a polyp of any size remained unchanged.

Alternative scenario 2 – add costs for OC and CTC complications and CTC extra-colonic findings

We estimated the 2015 Medicare cost per screening of complications following OC to be $96 and $20 for OC with and without biopsy, respectively, and the 2015 Medicare cost of complications and extra-colonic findings following CTC to be $131. We developed this by trending to 2015 costs reported in published literature [50]. OC has more complications than CTC and within OC, OCs with biopsies have substantially more complications than OCs without biopsies [51, 49, 47].

OC complication costs

In a Medicare population, inpatient hospital and emergency room costs for complications following OC were $48 per OC for 2007 as published in Leffler, 2010 [50]. We trended these costs to 2015 using an annual trend rate of 3% (for 8 years) to arrive at a 2015 cost of $61 per OC. The ASGE Complications of Colonoscopy – Guideline, 2011 [51] reported that 85% of complications are attributable to OCs with polypectomies (biopsies). We used this percentage and our finding that 54% of colonoscopies have biopsies, to calculate OC complication costs of $20 for OCs without biopsy and $96 for OCs with biopsy.

CTC complication and extra-colonic finding costs

Although costs estimates for CTC complications do not exist, the Medicare Decision Memo for Screening CTC for Colorectal Cancer (2009) [62], states that complications may occur due to colonic distention which is a critical requirement for an optimal screening. Given that CTC is much less invasive than OC, with or without biopsy, we assumed 10% of the OC without biopsy complication cost ($20) for an average CTC complication cost of $2.

Per patient costs for follow-up diagnoses resulting from extra-colonic findings following a CTC screening were $99 in 2006 as published in Pickhardt, et al, 2008 [63]. We trended these costs to 2015 using an annual trend rate of 3% (for 9 years) to arrive at a 2015 cost of $129 per CTC.

Thus, we modeled complication and extra-colonic finding costs for CTCs at $131 ($129 + $2).

Note that if a CTC is followed by an OC with biopsy, the complication costs associated with OC with biopsy ($96) is also applicable, resulting in an average estimated complication/extra-colonic finding cost for 2015 of $226 for CTCs with follow-up OC.

Alternative scenario 3 – increase anesthesia use for OC

As of January 1, 2015, anesthesia services that are separately billed are no longer subject to Medicare cost sharing. With this change, the use of anesthesia may increase. In this scenario we assumed that 80% of OCs performed will have anesthesia billed separately, a 40% increase in the use and cost of anesthesia services compared to the 57% base scenario assumption.

Alternative scenario 4 – add costs for CTC shared decision making

Medicare announced February 2015 that it will cover CT lung cancer screening with the provision that the first screening must include a documented shared decision making consultation [53]. If Medicare adopts a similar approach for CTC screening (but not OC), two consultations may be required: the first for the screening and the second for the decision for follow-up OC if the patient has small polyps (6-9mm).

CMS has not yet published the coding or reimbursement for shared decision making and, thus far, does not currently pay for shared decision making. We assumed that shared decision making would be provided in an evaluation and management (E&M) visit for both CTC and the decision for follow-up OC or surveillance after the finding of small polyps. Given the lack of guidance from Medicare, we assumed a cost of shared decision making of $20 for CTC and an additional cost of $20 for follow-up OC if the patient has small polyps. This cost is the 2015 non-facility Medicare reimbursement ($20) for Current Procedural Terminology (CPT®) code 99211[1] [24]. See Table A.7 for a list of all E&M CPT codes and their corresponding 2015 non-facility Medicare reimbursement.

Table A.7 – 2015 Non-Facility Medicare Reimbursement for Evaluation and Management CPT Codes

CPT Code	CPT Code Description	2015 National Non-Facility Medicare Reimbursement
99201	Office or other outpatient visit for the evaluation and management of a new patient, which requires these 3 key components: A problem focused history; A problem focused examination; Straightforward medical decision making. Counseling and/or coordination of care with other physicians, other qualified health care professionals, or agencies are provided consistent with the nature of the problem(s) and the patient's and/or family's needs. Usually, the presenting problem(s) are self limited or minor. Typically, 10 minutes are spent face-to-face with the patient and/or family.	$ 43.98

[1] CPT® is a registered trademark of the American Medical Association.

CPT Code	CPT Code Description	2015 National Non-Facility Medicare Reimbursement
99202	Office or other outpatient visit for the evaluation and management of a new patient, which requires these 3 key components: An expanded problem focused history; An expanded problem focused examination; Straightforward medical decision making. Counseling and/or coordination of care with other physicians, other qualified health care professionals, or agencies are provided consistent with the nature of the problem(s) and the patient's and/or family's needs. Usually, the presenting problem(s) are of low to moderate severity. Typically, 20 minutes are spent face-to-face with the patient and/or family.	$ 75.08
99203	Office or other outpatient visit for the evaluation and management of a new patient, which requires these 3 key components: A detailed history; A detailed examination; Medical decision making of low complexity. Counseling and/or coordination of care with other physicians, other qualified health care professionals, or agencies are provided consistent with the nature of the problem(s) and the patient's and/or family's needs. Usually, the presenting problem(s) are of moderate severity. Typically, 30 minutes are spent face-to-face with the patient and/or family.	$109.05
99204	Office or other outpatient visit for the evaluation and management of a new patient, which requires these 3 key components: A comprehensive history; A comprehensive examination; Medical decision making of moderate complexity. Counseling and/or coordination of care with other physicians, other qualified health care professionals, or agencies are provided consistent with the nature of the problem(s) and the patient's and/or family's needs. Usually, the presenting problem(s) are of moderate to high severity. Typically, 45 minutes are spent face-to-face with the patient and/or family.	$165.90
99205	Office or other outpatient visit for the evaluation and management of a new patient, which requires these 3 key components: A comprehensive history; A comprehensive examination; Medical decision making of high complexity. Counseling and/or coordination of care with other physicians, other qualified health care professionals, or agencies are provided consistent with the nature of the problem(s) and the patient's and/or family's needs. Usually, the presenting problem(s) are of moderate to high severity. Typically, 60 minutes are spent face-to-face with the patient and/or family.	$208.45
99211	Office or other outpatient visit for the evaluation and management of an established patient that may not require the presence of a physician or other qualified health care professional. Usually, the presenting problem(s) are minimal. Typically, 5 minutes are spent performing or supervising these services.	$ 20.02
99212	Office or other outpatient visit for the evaluation and management of an established patient, which requires at least 2 of these 3 key components: A problem focused history; A problem focused examination; Straightforward medical decision making. Counseling and/or coordination of care with other physicians, other qualified health care professionals, or agencies are provided consistent with the nature of the problem(s) and the patient's and/or family's needs. Usually, the presenting problem(s) are self limited or minor. Typically, 10 minutes are spent face-to-face with the patient and/or family.	$ 43.98
99213	Office or other outpatient visit for the evaluation and management of an established patient, which requires at least 2 of these 3 key components: An expanded problem focused history; An expanded problem focused examination; Medical decision making of low complexity. Counseling and coordination of care with other physicians, other qualified health care professionals, or agencies are provided consistent with the nature of the problem(s) and the patient's and/or family's needs. Usually, the presenting problem(s) are of low to moderate severity. Typically, 15 minutes are spent face-to-face with the patient and/or family.	$ 72.94

CPT Code	CPT Code Description	2015 National Non-Facility Medicare Reimbursement
99214	Office or other outpatient visit for the evaluation and management of an established patient, which requires at least 2 of these 3 key components: A detailed history; A detailed examination; Medical decision making of moderate complexity. Counseling and/or coordination of care with other physicians, other qualified health care professionals, or agencies are provided consistent with the nature of the problem(s) and the patient's and/or family's needs. Usually, the presenting problem(s) are of moderate to high severity. Typically, 25 minutes are spent face-to-face with the patient and/or family.	$108.34
99215	Office or other outpatient visit for the evaluation and management of an established patient, which requires at least 2 of these 3 key components: A comprehensive history; A comprehensive examination; Medical decision making of high complexity. Counseling and/or coordination of care with other physicians, other qualified health care professionals, or agencies are provided consistent with the nature of the problem(s) and the patient's and/or family's needs. Usually, the presenting problem(s) are of moderate to high severity. Typically, 40 minutes are spent face-to-face with the patient and/or family.	$146.24

Source: CMS Physician Fee Schedule for 2015

Alternative scenario 5 – decrease maximum screening age

The base scenario sets the maximum screening age at 84 in order to be consistent with claims currently paid by Medicare as they pay for CRC screening for all ages 50 and over. However, the USPSTF recommends CRC screening until age 75, which is used in this scenario.

Alternative scenarios 6 and 7 – decrease/increase OC follow-up rate for CTCs with small polyp findings

In the base scenario we assume that 50% of patients with small polyps found during a CTC screening will elect to have a follow-up OC. We could find no literature on how many patients with small polyps will have follow-up OC polypectomy vs. CTC surveillance. The percentage may vary substantially by clinic and physician. In this scenario we decreased the OC follow-up rate from 50% to 25% in scenario 6 and increased it to 75% in scenario 7.

Alternative scenario 8 – Decrease rescreen years for both OC and CTC for screenings with small polyps

In the base scenario, the average number of years between screenings is 6 for OC and 5 for CTC. At this time, USPSTF and Medicare have not established recommendations for CTC rescreening, and literature indicates that many OC patients rescreen sooner than recommended by guidelines [46]. For this scenario, we set the number of years to rescreen at 3 for both OC and CTC.

Alternative scenario 9 – Increase rescreen years for CTC to match OC

In this scenario, we set the number of years between CTC screenings to 3, 6, 7 and 10 for large, small, diminutive and no polyps, respectively, to match the rescreen intervals of OC.
